# Use of operative laparoscopes in single-port surgery: The forgotten tool

**DOI:** 10.4103/0972-9941.72403

**Published:** 2011

**Authors:** Arjun Khosla, Todd A. Ponsky

**Affiliations:** Division of Pediatric Surgery, Rainbow Babies & Children’s Hospital, Case Western Reserve University, Cleveland, OH

**Keywords:** Operative laparoscope, pediatric, children, single site, single port

## Abstract

Single-port surgery is an emerging advancement in the field of minimally invasive surgery. Several different techniques and tools have been developed to decrease the invasiveness of various operations. Amongst these new developments, many general surgeons have overlooked an important tool, the operative laparoscope. These telescopes reduce the number of ports placed during minimally invasive operations by providing both visualization and operative channels to accommodate instruments. We have described several simple techniques that employ the operative laparoscope to reduce the number of incisions in laparoscopic surgery with good outcomes. Single-port surgery has been shown to be safe and effective and may someday replace traditional laparoscopy in the performance of minimally invasive operations.

## INTRODUCTION

Recently, single-port surgery has gained popularity in the field of minimally invasive surgery (MIS). Numerous techniques have evolved in an effort to decrease the invasiveness of operations. These techniques aim to reduce the number and length of incisions leading to better cosmesis, decreased pain, quicker recovery and reduced cost. However, the introduction of single-port surgery brought with it the development of many new tools and devices. Most of these tools are not only complex, but also costly. They include multi-port and low-profile trocars, extra-long and articulating telescopes and articulating hand instruments. With the development of these new, elaborate instruments, most surgeons have forgotten about one of the most basic operative tools in the surgeon’s arsenal, the operative laparoscope. There are numerous versions of the operative laparoscope, depending in which specialty of surgery it is being used. While operative laparoscopes are used often in urology, neurosurgery and gynaecology, most general surgeons do not use them. We describe the use of operative laparoscopes to minimize the number of incisions in laparoscopy, and even in performing single port-surgery.

Similar to single-port surgery, several other successful techniques have been developed to reduce the invasiveness of laparoscopic operations, including two-port surgery, laparoscopic-assisted approaches and natural orifice transluminal endoscopic surgery (NOTES). The two-port and laparoscopic-assisted techniques use an operative laparoscope through one port, while an accessory port is placed to assist in retraction.[1] For example, Rothenberg *et al*. have described the use of a “modified” single-port technique, using an additional 3-mm instrument to aid in dissection and triangulation.[2] NOTES involves performance of the entire procedure through a natural orifice, such as the mouth or vagina, by using multichannel endoscopes. However, some have questioned whether the improved cosmesis is worth the risk of visceral injury. In addition, these techniques require specialized equipment. To eliminate these disadvantages of NOTES, the same technology using telescopes with operative channels can be applied to single-port surgery, using an operative laparoscope, with very good outcomes.

## 8-MM/10-MM OPERATIVE LAPAROSCOPE

### Appendectomy

A traditional laparoscopic appendectomy involves three trocars, requiring three separate incisions. A 10-mm umbilical port is placed for the laparoscope, and two additional 5-mm ports are placed for retraction and dissection. Recently, several have described performing single-incision appendectomies. This usually involves three trocars through the umbilicus. However, many paediatric surgeons are now utilizing an operative laparoscope to perform truly single-port appendectomies. Various techniques have been described, both intracorporeal and extracorporeal. Intracorporeal methods often use three trocars through the umbilicus, or require a method, such as a transdermal suture, to elevate the appendix for dissection and ligation.[3,4]

**Figure 1:**
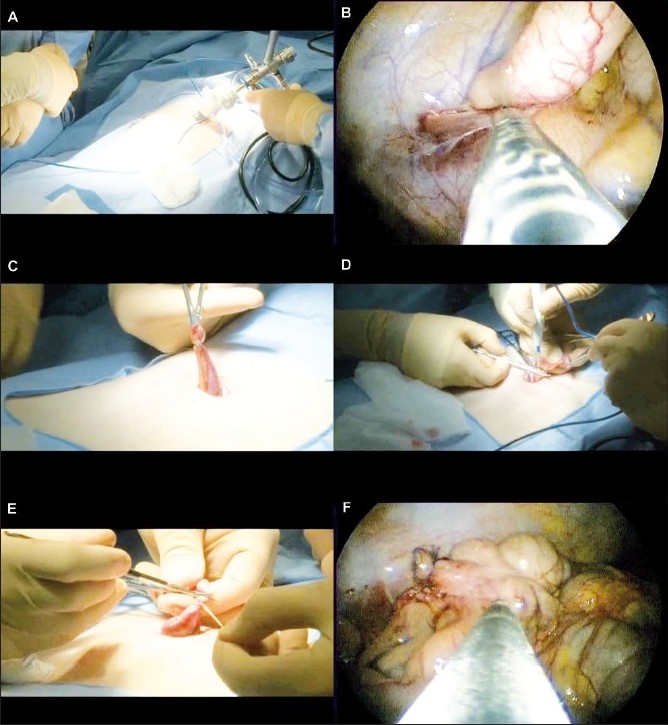
Single-Port Laparoscopic Appendectomy. A) The laparoscope is inserted through the umbilicus. B) A Maryland dissector is used to free the appendix from attachments. C) The appendix is pulled through the umbilicus. D) The mesoappendix is divided. E) The appendix is ligated and divided. F) The laparoscope is reinserted to ensure the appendiceal stump is short and not bleeding.

**Figure 2:**
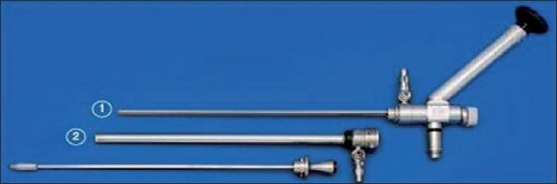
The Frazee operative laparoscope (KARL STORZ, Tuttlingen, Germany) is a 6° 8-mm straight-viewing Hopkins laparoscope, 30-cm length, with a 5-mm working channel. The laparoscope’s short length allows for the use of standard laparoscopic instuments.

The extracorporeal technique involves the insertion of one 10-mm trocar through the umbilicus and use of an 8-mm operative laparoscope with a 5-mm working channel. The appendix is dissected free using a Maryland dissector or hook cautery, inserted through the operative laparoscope channel. The tip of the appendix is grasped and brought out through the umbilicus. The mesoappendix and appendix are then divided extracorporeally. The scope is then reinserted to ensure that the appendiceal stump is short and there is no bleeding[5] [Figure 1]. In our practice, we typically use a modified prototype Frazee operative laparoscope, which is a 6° 8-mm straight-viewing Hopkins laparoscope, of 30-cm length, with a 5-mm working channel [Figure 2]. The short length allows for the use of standard laparoscopic instruments. Unfortunately, this scope is not commercially available. However, there are many similar laparoscopes that are available. An alternative telescope for this technique is the straightforward operative telescope (KARL STORZ, Tuttlingen, Germany), which has a 10-mm diameter, 27-cm length and 6-mm working channel. This laparoscope accommodates 5-mm × 42-cm instruments. Ideally, shorter operative laparoscopes that could accommodate standard length 36-cm instruments would be ideal, but are not yet available commercially.

Single-port, transumbilical, extracorporeal appendectomies have been well described in the literature and have been shown to be safe and effective alternatives to the traditional laparoscopic appendectomy with a comparable post-operative complication rate.[6–15] With new instrumentation, this procedure may now be easier and safer. The technique, first described in adults by Pelosi in 1992 and in children by Begin in 1993, combines elements of traditional and laparoscopic surgery, which gives the surgeon the advantages of both approaches. The patient benefits from the decreased invasiveness of laparoscopic surgery and the hospital benefits from the reduced costs of open techniques. This method may be more cost-effective by decreasing the amount of equipment, operating room staff and operating room time required to complete the procedure. In addition, the economy of motion is improved, with only one incision to open and close, one instrument placed through the working port and one or two ties placed on the appendix.[6]

When compared to the single-port method through the right iliac fossa, the umbilical approach may be advantageous, especially in children. In small children, the iliac vessels may be very close to the anterior abdominal wall, risking injury if the laparoscope is placed there. In addition, access to the abdominal cavity is easier and faster through the umbilicus where there are only two layers, skin and linea alba. For this reason single-port surgery at the iliac fossa is contraindicated in obese patients to avoid leaving a long appendiceal stump. Abdominal exploration is also easier with the telescope at the umbilicus because placement in the iliac fossa may be too close to the ileocecal area for full comprehension of the disease.[9]

The limitations of this procedure are inherent to the technique. As with any new technique, there is a learning curve, but the operation is learned quickly and easily. Also, dense adhesions, perforated appendicitis, generalized peritonitis or a retrocecal appendix may require the addition of an accessory port or conversion to a traditional laparoscopic appendectomy. The conversion rate is likely higher in adults than in children because the distance between the cecum and umbilicus is shorter and the abdominal wall is more supple in children.[9]

### Ovarian cystectomy

Ovarian torsion is a surgical emergency that occurs unilaterally in a pathologically enlarged ovary. Commonly, torsion is caused by benign ovarian cysts greater than 5-cm in size, which create a fulcrum around which the ovary revolves. Delay in diagnosis or treatment leads to ovarian infarction and necrosis. Standard laparoscopic treatment of ovarian torsion involves three-port laparoscopy (one 10-mm port and two 5-mm ports), with detorsion of the ovary, drainage of the cyst and possible oophoropexy.

The use of single-port laparoscopy to detorse the ovary and treat ovarian cysts, while sparing the ovarian tissue itself, has been described in the literature.[16–18] These methods have been shown to be feasible, safe, effective and provide minimal post-operative pain and bleeding with improved cosmesis. Although we have not performed a detorsion using an operative laparoscope, we have performed diagnostic laparoscopy and drainage of ovarian cysts for presumed torsion. We utilized a 10-mm trocar through the umbilicus and inserted the operative laparoscope through the trocar. The ovarian cyst was identified and drained. There was no torsion and the rest of the abdomen was explored for other causes of pain.

Limitations of this technique are common to other procedures involving the operative laparoscope. There are fewer degrees of freedom between the instruments, inside and outside the abdominal cavity, which limits the surgeon’s movements. To alleviate this issue, some have suggested the use of an intrauterine device to manipulate the position of the uterus, an angled laparoscope and a combination of standard 36-cm and long 42-cm instruments.[17]

### Decortication for empyema

We have also used the 8-mm operative laparoscope to treat empyema. In one child, who had developed an empyema secondary to pneumonia, an incision was made in the 5^th^ intercostal space and the operative laparoscope was inserted. The fluid was aspirated and the lung was decorticated to achieve full expansion. The peel was removed using a Maryland grasper through the scope. A chest tube was then inserted through the incision.

### Lung wedge resection and pleurodesis

A 16-year-old with a recurrent spontaneous pneumothorax was also treated with an 8-mm operative laparoscope. The scope was inserted through the 5^th^ intercostal space. The lung was found to have a bleb at the upper lobe apex. An 0-PDS Endloop (Ethicon, Cincinnati, OH) was placed in the same incision alongside the scope. The bleb was grasped through the endoloop and the endoloop was cinched down below the bleb. A second endoloop was placed in a similar fashion. A scissors was inserted through the scope and the bleb resected. Talc was then injected through the scope into the pleural space. A chest tube was then placed through the incision.

## 4-MM OPERATIVE LAPAROSCOPE

### Gastrostomy

There are numerous techniques available for placement of a gastrostomy in a child. The most popular standard techniques are either the percutaneous endoscopic gastrostomy (PEG) or the laparoscopic gastrostomy. The PEG is based on the principle of endoscopic approximation of the stomach and peritoneum with blind percutaneous entrance into the abdominal cavity, through the peritoneum, and into the stomach. The putative advantage of the laparoscopic gastrostomy is that the stomach and abdominal wall can be visualized directly, thus limiting the chance of inadvertent injury to the colon, liver, oropharynx or oesophagus. In addition, PEG placement can be difficult to perform and is associated with more complications in patients with neurological impairment, foregut anomalies, craniofacial anomalies or prior abdominal operations. Overall, the morbidity of PEG placement is 5-13%.[19,20]

**Figure 3:**
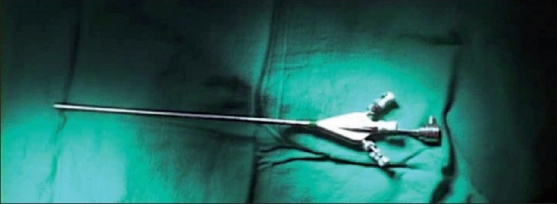
The 4-mm operative hysteroscope (KARL STORZ, Tuttlingen, Germany) is able to fit through a 5-mm trocar and has a 1-mm operative channel, through which a wire biopsy forceps fits.

**Figure 4:**
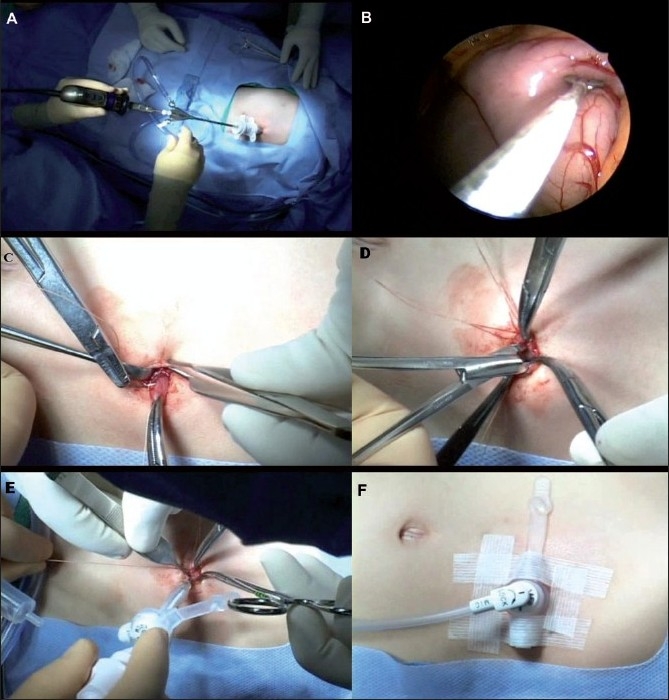
Single-port Laparoscopic Gastrostomy. A) The hysteroscope is placed through a trocar in the left upper quadrant. B) The greater curvature of the stomach is grasped. C) The stomach is then brought out through the trocar site. D) A gastrostomy is created. E) The gastrostomy is matured to the fascia and a primary gastrostomy button is placed over a Veress needle. F) Once position is confirmed, the sutures are tied and the button is secured with Steri-Strips.

The traditional laparoscopic gastrostomy involves two skin incisions: one for the telescope and one for the grasper. Therefore, although the laparoscopic technique is associated with less risk of injury, it is more invasive and time-consuming than PEG. Some techniques for single-incision laparoscopic gastrostomy, which is less invasive than traditional laparoscopy while still allowing for direct visualization, employ multiple trocars through the same incision. However, Kawahara *et al*. described the benefits of using an operative laparoscope to perform a single-port laparoscopic gastrostomy.[21] Similarly, we utilize a 4-mm operative hysteroscope (KARL STORZ, Tuttlingen, Germany) to perform the single-port laparoscopic gastrostomy [Figure 3]. The operative laparoscope is able to fit through a 5-mm trocar and has a 1-mm operative channel, through which a wire biopsy forceps fits. The operation begins by inserting a 5-mm trocar through a left upper quadrant incision and inserting the hysteroscope through the trocar. The stomach is identified by asking the anaesthesiologist to insufflate it with air. Once the stomach is seen to increase in size, the greater curvature is grasped at the appropriate location and brought out through the trocar site. The gastrostomy is then matured to the fascia and a primary gastrostomy button is placed[5] [Figure 4].

We previously described a similar single-port technique for laparoscopic gastrostomy that employed a 4-mm operative bronchoscope.[22] However, this technique was associated with some problems. The bronchoscope provided poor visibility, mostly due to the instrument’s optics and the fact that half of the screen was obstructed by the grasper. Furthermore, because the bronchoscope was not perfectly round, it was difficult to maintain a tight seal in the trocar, which made it difficult to maintain pneumoperitoneum. The poor insufflation, along with poor optics of the scope, made it difficult to identify the optimal location on the stomach where the gastrostomy should be placed. We found that the 4-mm operative hysteroscope provided much better optics for this technique with no leakage of air. This instrument fits nicely in the 5-mm trocar, but the 4-mm size of the instrument prevents insufflation through the trocar, so the insufflation tubing needs to be moved to the scope once it is inserted.

### Inguinal hernia

Laparoscopic hernia repair offers many advantages, including improved cosmesis, decreased pain and quicker recovery. In addition, it allows for the ability to evaluate the contralateral side for a patent processus vaginalis, avoidance of access trauma to the vas deferens and gonadal vessels, and decreased operation length after the initial learning curve. Standard laparoscopic hernia repair involves three trocars: one at the umbilicus and two lateral ports for hand instruments. A less invasive laparoscopic technique for paediatric hernia repair described by Ozgediz, *et al*. involves closing the hernia sac with a subcutaneous endoscopically assisted ligation (SEAL) technique.[23] This technique involves transperitoneal endoscopic visualization of the internal ring with a 2.7-mm scope, allowing a safe high ligation of the hernia sac at the internal ring by manipulation of the needle and suture percutaneously. This method eliminates the need for lateral ports. However, in certain cases, an additional instrument is placed to manipulate the hernia sac for better visualization.

Our technique once again utilizes the 4-mm operative hysteroscope, this time through the umbilicus. This allows for hernia sac manipulation without the need for an additional port. The aim of such manipulation is to maximize visualization of the peritoneum in order to avoid injury to the vas deferens and gonadal vessels. We find that pulling on the peritoneum moves the operative hysteroscope, thus moving the internal ring out of view. To alleviate this issue, we employ the grasper and use its jaws to spread, rather than pull, the peritoneum to maximize visualization. Under direct visualization, the internal ring is encircled by passing a nonabsorbable suture from one side, excluding the vas deferens and gonadal vessels, to the other. This method of using the operative hysteroscope is currently under investigation and, although not yet perfect, it is promising.

## CONCLUSION

Single-port surgery represents a natural progression of MIS towards fewer incisions, decreasing costs and improving patient outcomes. Various techniques have been developed to reduce the invasiveness of existing operations. However, many general surgeons have overlooked an important tool, the operative laparoscope. These telescopes reduce the number of ports placed during minimally invasive operations by providing both visualization and operative channels to accommodate instruments. We have described several simple techniques that employ the operative laparoscope to reduce the number of incisions in laparoscopic surgery with good outcomes. These same principles can be applied to any operation in which the telescope may lie parallel to the laparoscopic instruments. Single-port surgery has been shown to be safe and effective and may someday replace traditional laparoscopy in the performance of minimally invasive operations.
